# Environmental Susceptibility of the Sperm Epigenome During Windows of Male Germ Cell Development

**DOI:** 10.1007/s40572-015-0067-7

**Published:** 2015-09-11

**Authors:** Haotian Wu, Russ Hauser, Stephen A. Krawetz, J. Richard Pilsner

**Affiliations:** Department of Environmental Health Sciences, School of Public Health and Health Sciences, University of Massachusetts Amherst, 149 Goessmann, 686 North Pleasant Street, Amherst, MA 01003 USA; Department of Environmental Health, Harvard T.H. Chan School of Public Health, Harvard University, Building I 14th Floor, 665 Huntington Avenue, Boston, MA 02115 USA; Department of Epidemiology, Harvard T.H. Chan School of Public Health, Harvard University, Building I 14th Floor, 665 Huntington Avenue, Boston, MA 02115 USA; Department of Obstetrics and Gynecology, C.S. Mott Center for Human Growth and Development, Wayne State University School of Medicine, 275 East. Hancock, Detroit, MI 48201 USA

**Keywords:** Sperm, Epigenetics, Environmental exposures, DNA methylation, Histones, Non-coding RNA, miRNA, Protamines, Spermatogenesis, Male germ cells, Nutrition

## Abstract

Male germ cells require multiple epigenetic reprogramming events during their lifespan to achieve reproductive capacity. An emerging body of compelling data demonstrates that environmental exposures can be embodied within the developing male germ cell as epigenetic marks. In turn, these epigenetic marks can impart information at fertilization to affect the trajectory of offspring health and development. While it is recognized that in utero epigenetic reprogramming of male germ cells is a particularly susceptible window to environmental exposures, other such windows exist during germ cell development. The objective of this review is to discuss epigenetic reprogramming events during male germ cell development and to provide supporting evidence from animal and human studies that during specific periods of development, germ cells are susceptible to environmentally induced epigenetic errors. Moving forward, the nascent field of sperm epigenetics research is likely to advance our understanding of paternal environmental determinants of offspring health and development.

## Introduction

Spermatozoa have been traditionally considered vehicles for the sole delivery of the paternal genome to oocytes upon fertilization. In this context, paternal contributions to offspring phenotype are strictly limited to germline genetic information without the ability to impart environmental information that is encountered during the life course. However, a growing body of compelling data demonstrates that certain environmental exposures can be embodied within the developing male germ cell without altering the germline genetic information and, in turn, can affect the offspring phenotype.

Epigenetics is the study of semipermanent, mitotically heritable and, in germ cells, meiotically heritable changes in gene expression that primarily result from modifications of chromatin structure, rather than changes in the underlying DNA sequence [1]. The three major mechanisms of epigenetics are DNA methylation primarily within CpG dinucleotides [2], a host of modifications to histone tails [3], and non-coding RNAs (e.g., microRNAs and long non-coding RNAs) [4]. In concert, these epigenetic mechanisms control chromatin structure to confer cell-specific gene expression.

In humans, male germs cells do not attain reproductive capacity until the second decade of life. Despite this long latency period, male germ cells begin development early in fetal life and, upon sex determination, embark on a remarkable journey of cellular differentiation and morphological changes to prepare for its sole purpose—the propagation of its genome. During development, male germ cells progress from primordial germ cells (PGCs), diploid spermatogonia to haploid spermatozoa that involves stage- and testis-specific gene expression, mitotic and meiotic divisions, and chromatin remodeling that is unique only to sperm [5, 6]. To undergo these transformations, stage-specific epigenetic reprogramming is required in addition to more modest, but still significant, epigenetic changes that gradually progress germ cell phenotype toward reproductive capacity. As the epigenome allows considerable cellular plasticity, epigenetic changes across the many stages of male germ cell development represent windows of susceptibility by which environmental exposures can sculpt the epigenetic landscape.

In this review, we identify and discuss multiple windows of susceptibility during mammalian male germ cell developmental in which dietary and toxicant exposures have been shown to influence sperm epigenetics as well as offspring phenotype in animal models and humans.

## Windows of Male Germ Cell Development

### In Utero Period and Primordial Germ Cells

**Fig. 1:**
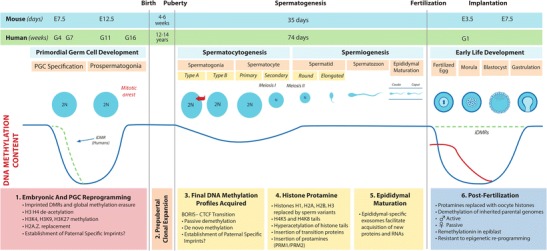
Windows of susceptibility during male germ cell development. (*1*) Primordial germ cells (PGCs) arise from proximal epiblast (E7.5 in mouse and G4 in humans) and undergo clonal expansion as they migrate and colonize the genital ridge. Epigenetic remodeling of histone and DNA methylation marks of PGCs are essential to achieve totipotency for sex-specific epigenetic programming. In mice, comprehensive loss of methylation in PGCs occurs (around E13.5) passively via Uhrf1 silencing and actively via Tet proteins to remove imprinted marks; while in humans, the first wave occurs around G7 with the second wave, via loss of H3K27me3, to erase imprinted marks at G11. Afterward, de novo methylation occurs via Dnmt3a, Dnmt3b, and the non-catalytic Dnmt3l. Histone modifications after PGC specification include hypoacetylation of H3 and H4; hypermethylation of H3K4, H3K9, and H3K27; and replacement of the histone variant, H2A.Z. (*2*) After birth, rapid expansion of spermatogonia occurs in mice; however, after an initial clonal expansion, germ cells remain most dormant with intermittent expansion, most notable a few years before puberty upon awakening of the HPG axis. This prepubertal clonal expansion may be susceptible to environmental exposures as indicated by epidemiologic evidence. (*3*) Initiated at the onset of puberty by the activation of HPG axis, spermatogenesis occurs in the seminiferous epithelium and is supported by mitotically inactive Sertoli cells. Final DNA methylation patterns, including imprinted domains, are acquired possibly via CTCF–BORIS switch during spermatocytogenesis. Also, histone variants begin to be incorporated. (*4*) During the first stage of spermiogenesis, extensive chromatin remodeling occurs via the histone-protamine exchange, with acetylation of histone, insertion and removal of transition proteins, and then insertion of protamines 1 and 2. Approximately 90 and 99 % of histones are replaced with protamines in humans and mice, respectively. (*5*) During epididymal maturation, the last stage of spermiogenesis, germ cells become motile and exosomes shuttle proteins and ncRNA to mature spermatozoa. (*6*) Shortly after fertilization, the two parental genomes are demethylated in an asymmetrical manner: the paternal genome is actively depleted of DNA methylation, while the maternal genome (shown in read), which harbors substantially less DNA methylation than sperm, undergoes a passive loss of DNA methylation that is characterized by a dilution effect as a result of the lack of maintenance of DNA methylation over multiple cleavage divisions. Demethylation is not complete as imprinted genes intra-cisternal A particles (IAPs) and heterochromatin regions around centromeres largely escape this demethylation event. Sperm protamines are replaced with oocyte histones with hours of fertilization. Windows of susceptibility during male germ cell development figure; (2015), by J. Richard Pilsner. Made available under Creative Commons Attribution 4.0 License

Primordial germ cells (PGCs) arise from the proximal epiblast with a population of <50 cells and undergo clonal expansion as they migrate and colonize the genital ridge, the precursor to the gonads [7] (Fig. 1). As PGCs are derived from cells of the epiblast, which have begun on a course of somatic fate, epigenetic reprogramming is essential to re-establish totipotency for sex-specific epigenetic programming of germ cells. The loss of genome-wide methylation occurs passively during the rapid proliferation of PGCs. Although the maintenance DNA methyltransferase 1, DNMT1, is readily expressed in PGCs, its essential cofactor, Uhrf1, is not, resulting in the loss of maintenance of methylation during cell divisions [8]. Imprinted-specific differential methylated regions (iDMRs), which are methylated in a parent-of-origin manner and have escaped epigenetic reprogramming shortly after fertilization, follow slower kinetics requiring active demethylation via Tet proteins in mice [9]. In humans, a second wave of reprogramming in PGCs occurs several weeks later to erase imprinted marks via histone remodeling, most notable depletion of H3K27me3 and removal of the histone variant, H2A.Z [10]. At the end of methylation erasure, global levels of methylation of male PGCs are estimated at 16.3 % compared to the 70 % methylation in the embryo [11]. The lack of complete erasure is mostly due to the resistant nature of intra-cisternal A particles (IAPs), a class of retrovirus-like transposons, and their proximal genes, to demethylation, which bestows a potential mechanism for epigenetic inheritance [12]. The majority of methylation is re-established in mitotically arrested type A spermatogonia prior to birth and is fully resolved postnatally during spermatogenesis [13].

#### Nutritional Manipulation

Given the extensive reprogramming that occurs in PGCs to redefine their epigenetic landscape in a sex-specific manner, environmental exposures in animal models during this period have been shown to induce intergenerational and transgenerational effects through the sperm epigenome. Severe in utero caloric restriction during the window of re-acquisition of DNA methylation in mouse (E16.5) led to differential methylated regions (DMRs) in F1 sperm mainly at intergenic regions and CpG islands, which were also reported to associate with regions of histone retention [14•]. While both F1 and F2 male mice exhibited metabolic-related disorders, DMRs of F1 sperm did not persist in somatic tissue of F2 males [14•]. Interestingly, the expression of nearby metabolic genes was altered in F2 males, indicating that although sperm DMRs were lost, other epigenetic mechanisms, not measured in this study, could persist to influence F2 gene expression [14•]. These results are in contrast to another study in which in utero caloric restriction resulted in the transmission of altered DNA methylation of a lipogenic gene, Lxra, in F1 sperm to F2 somatic tissues [15]. Additionally, streptozotocin-induced gestational diabetes altered the expression of imprinted genes, IGF2 and H19, in F1 sperm and F2 pancreatic islets [16] and increased Peg3 DNA methylation in F1 sperm [17].

In an intriguing study in male mice, life-long (i.e., in utero and adult) deficiency in folate, a key component of one-carbon metabolism that facilitates the transfer of methyl groups for DNA and histone methylation reactions, resulted in craniofacial and musculoskeletal birth defects in their offspring [18]. Genome-wide analyses of sperm from folate-deficient animals in adulthood identified 57 DMRs, none of which was associated with iDMRs, but rather, they were associated with genes related to cancer, diabetes, and neurological diseases. Moreover, global mono- and tri-methylations at H3K4 and H3K9 were also reduced in folate-deficient F1 sperm [18]. While in the placenta, over 300 genes were differentially expressed; however, only two associated with sperm DMRs, suggesting that other epigenetic modifiers such as sperm H3 methylation were involved. It must be noted, however, that since exposure was life-long, it is difficult to discern the timing of germ cell development (e.g., PGCs or spermatogenesis) in which folate deficiency induced these observed epigenetic effects.

#### Environmental Toxicants

Skinner and colleagues have repeatedly demonstrated environmental toxicant-induced transgenerational effects through the paternal germ line in outbred rats. In utero exposure to chemicals exhibiting endocrine-disrupting characteristics, such as vinclozolin [19–22], DDT [23]; 2,3,7,8-tetrachlorodibenzo[p]dioxin (TCDD) [24, 25]; the jet propellant, JP8 [24, 26]; pesticide mixture of permethrin and DEET [24, 27]; and plastic mixture of bisphenol-A, (BPA), bis(2-ethylhexyl)phthalate (DEHP), and dibutyl phthalate (DBP) [24, 28] all elicited DMRs in F3 sperm without any additional exposures in subsequent generations. Interestingly, the DMRs, which were mostly intergenic, displayed little overlap between exposures [23, 24], indicating the lack of specificity of environmentally induced DMRs in male germ cells. Moreover, vinclozolin exposure at a similar dose and timing produced no overlapping DMRs in sperm of F3 rats [21] and mice [20], demonstrating again that the sperm epigenome may be programmed by environmental toxicants in a stochastic fashion.

In contrast to the faithful inheritance of the transgenerational effects reported above, other studies have reported that in utero exposure to pesticides, vinclozolin and methoxychlor, modified methylation of iDMRs in F1 sperm, but a trend toward recovery was observed starting in the F2 sperm and continued through the F3 [29, 30]. Similarly, in utero exposure to the endocrine disruptors, vinclozolin, BPA, or DEHP, in mice resulted in DNA methylation changes in F1 prospermatogonia, but these changes did not persist into the F2 germline [31••]. Most recently, in utero exposure to vinclozolin was found to alter the expression of microRNAs (miRNAs), miR-23b, miR-21, and *let-7*, in F1–F3 PGCs; however, no prominent changes in DNA methylation were observed in either F1 PGCs or mature sperm [32•].

It is currently unclear from the above studies whether the observed environmentally induced DMRs in sperm are direct effectors of offspring programming or they are themselves biological intermediates for other epigenetic modifiers [14•, 18], such as unmeasured histone modifications and/or altered non-coding RNA expression. Alternatively, these DMRs may act as non-causal markers of exposures, such that environmental exposures may operate through other pathways to induce adverse offspring health. As a consequence of the difficulty of conducting life course studies in humans, there is currently no data that we are aware of on the associations between in utero exposures and adult sperm epigenetic endpoints.

### Infancy and Prepubertal Periods

The timing of postnatal testicular development varies considerably among mammalian species with a marked distinction between rodents and higher primates [33] (Fig. 1). In laboratory rodents, testicular development begins a few days after birth in which mitotically arrested prospermatogonia resume clonal expansion resulting in an estimated 30-fold increase in spermatogonia prior to puberty [34]. In contrast, humans have a long latency period between birth and puberty, whereby after the first few months of postnatal life, referred to as mini-puberty, steroidogenic activity and testicular development are thought to remain quiescent until the onset of puberty [35].

This notion of inactive testicular development in childhood was largely driven by palpation and Prader’s orchidometer measures that detected no change in testicular volume until the onset of puberty [36]. However, employing more sensitive methods, such as stereological measures from testes obtained after autopsy, data indicate that testes, despite displaying no outwardly changes in size, are actively developing organs during infancy [37, 38]. For example, during the first 10 years of life, stereological measures revealed that testicular volume tripled with increases in seminiferous tubule length and the number of spermatogonia and Sertoli cells [37]. Another study reported that germ cell proliferation is not linear with age but may occur in waves, such that, during periods from 3 to 8 years and at 10 years to the onset of puberty, experienced marked proliferative activity [38]. This proliferation has been proposed to be related to transient awakening of the hypothalamus–pituitary–gonadal (HPG) axis during childhood [39] and to a more pronounced awakening around 2 years before puberty onset, also known as the slow growth period [40–42]. Thus, the HPG axis during infancy, and especially prior to puberty, may be activated to “prime” spermatogonia proliferation prior to full activation at puberty.

This “priming” of spermatogonia proliferation provides a biological explanation for the epidemiologic data associating prepubertal environmental exposures with male germ line effects [43]. In Seveso, Italy, acute high TCDD exposure from a chemical plant accident during infancy/prepuberty was associated with reduced sperm concentration and motility, while the opposite was observed with exposure around puberty [44]. Moreover, a reduction in estradiol and an increase in FSH were observed in both groups; however, no changes in hormone levels or sperm quality were observed among TCDD-exposed adults [44]. In support of the observed time-dependent effects of TCDD, using population data in Överkalix, Sweden, studies reported that the grandchild experienced shorter survival and greater risk of diabetes mortality if the paternal grandfather experienced at least one “good” harvest during the ages of 9 to 12 and longer survival and decreased risk of diabetes if the paternal grandfather experienced at least one “poor” harvest during the same age period [45–47]. More recently, male, but not female, offspring of men who were smoking before the age of 11 were found to have an increased BMI at age 7 and increased waist circumference and fat mass by age [46], which persisted through the latest follow-up at age 17 [48]. Offspring of mothers who reported smoking before the age of 11 showed no increase in BMI up to age 17 [48]. Together, these studies, while they lack sperm epigenetic data, provide compelling data indicating that the prepubertal period, a time in which the HPG axis begins to awaken to drive spermatogonia proliferation, is a sensitive period in which environmental exposures may target the epigenetic programming of germ cells. Epidemiologic studies are needed to confirm these observational studies by demonstrating that environmental exposures during the prepubertal period are associated with sperm epigenetics across generations.

### Spermatogenesis in Adulthood

To date, the majority of experimental research in animals has focused on environmental exposures during in utero epigenetic reprogramming of PGCs with little regard to other susceptible periods occurring in the adult. Spermatogenesis, the final process of germ cell development that entails the progression from diploid spermatogonia to haploid spermatozoa, requires dynamic epigenetic reprogramming for the production of viable sperm for fertilization (Fig. 1). In humans, spermatogenesis is estimated to take around 74 days (around 35 days in mice) to produce mature spermatozoa from undifferentiated spermatogonia, and it can be divided into two sequential processes: spermatocytogenesis, which includes spermatogonial proliferation and differentiation through mitosis to produce spermatocytes and meiosis I and II to produce round spermatids, and spermiogenesis, in which differentiation and maturation of spermatids occur without further cellular division (Fig. 1). In the end, 32 spermatozoa are produced from one type B spermatogonium in humans, which is in great contrast to rodents where premeiotic cell divisions are intense, such that one spermatogonium has the potential to produce 4096 spermatids [33]. This dramatic difference in clonal expansion of male germ cells among man and rodents may beget caution in the interpretation of rodent data. For example, if an epigenetic error such as DNA methylation occurs in the first few cell divisions in humans, this error, if not corrected, would propagate to affect only a few spermatozoa in a large pool, compared to the same scenario in mice where this error is likely to be more pronounced.

Acquisition and loss of methylation have also been reported during spermatocytogenesis [13, 49]. In adult mice, passive demethylation, likely occurring during spermatogonial cell divisions, was found to be enriched in interspersed repeat sequences, while methylation acquisition was observed in the pachytene stage of primary spermatocytes and was enriched in non-repeat sequences located within or flanking gene bodies as well as in paternal iDMRs [13]. The mechanism of this targeted resetting of DNA methylation during spermatocytogenesis may be linked with the expression of BORIS [49], a testis-specific protein paralogous to the insulator protein of imprinted marks, CTCF. Interestingly, BORIS and CTCF were expressed in a mutually exclusive manner during spermatogenesis in mice and humans [49]. The proposed model suggests that BORIS is upregulated in primary spermatocytes and associates with demethylases that erase methylation marks, and once CTCF is reactivated (and BORIS removed), targeted de novo methylation of paternal imprints and other regions is initiated in postmeiotic cells [50], which contradicts previous findings [13], likely due to methodological differences in methylation detection. Furthermore, age-dependent intra-individual alterations in sperm DNA methylation have been reported, indicating that sperm methylation can be modified throughout the adulthood [51]. Taken together, these data signify that spermatocytogenesis is an important developmental period that shapes DNA methylation profiles of mature spermatozoa.

After acquisition of final DNA methylation profiles, spermatids enter spermiogenesis, a multistep developmental window of global reorganization of chromatin [52]. Starting during meiosis, the canonical histones, H1, H2A, H2B, and H3, are replaced by testis-specific variants, which decrease the stability of nucleosomes [53, 54]. Next, hyperacetylation of histone tails occurs, most notably at H4K5 and H4K8, which “relaxes” nucleosomes to further enhance histone destabilization [52]. Brdt, a testis-specific protein harboring two bromodomains capable of specifically recognizing acetylated histones, is then recruited to H4K5 and H4K8 acetylation to facilitate histone removal [55, 56]. Transition nuclear proteins, TNP1 and TNP2, then displace histones and are themselves replaced with the protamine proteins, protamine 1 and protamine 2 (PRM1 and PRM2), which are typically found in equal proportions [57]. Protamine packaging of DNA restricts transcriptional activity and therefore has been proposed as a non-traditional form of epigenetic regulation unique to sperm cells [6]. It also is critical to enhance motility and safeguard the paternal genome from the harsh environment soon to be encountered in the epididymis and female reproductive tract [58].

This histone–protamine exchange; however, is not complete, such that an estimated 10 and 1 % of histones in humans and mice, respectively, are retained in mature sperm [59, 60]. Histone retention is also not randomly distributed throughout the genome, suggesting that they may play a form of postfertilization epigenetic regulation. Several studies using human and mouse sperm report that histone retention is enriched in regulatory regions of developmental and imprinted genes [59, 61–63]. However, two recent studies contradict these findings showing that nucleosomes were generally not located in promoter regions including developmental promoters but rather in gene-poor regions [64, 65]. Additional work is needed to resolve these opposing findings before a definitive role for sperm nucleosomes, as well as their histone modifications, is assigned to embryo development.

Upon exiting the testes, spermatozoa are morphologically transformed but are immotile and lack fertilization potential. Sperm maturation occurs through sequential modifications within distinct microenvironments during the 1–2-week transit through of the epididymis, which is estimated to be 6–7 m long in humans [66, 67]. Additionally, epididymal-specific exosomes (“epididymosomes”) are reported to act as carriers of somatic proteins and RNAs to sperm [68, 69•, 70]. Interestingly, epididymal secretions are regulated by androgens [71, 72], indicating that environmental factors that disrupt endocrine signaling may impact sperm procurement of exosomal proteins and RNAs [73]. Thus, while epididymal sperm maturation is directed at the acquisition of fertilization potential, exosomal shuttling may also provide the final opportunity for sperm to “epigenetically match” their current environment prior to fertilization. To our knowledge, no study has examined the direct effect of environmental exposures in the epididymis on the sperm epigenome; however, recently developed model systems may provide future insights [69•].

#### Nutritional Manipulations

Along with in utero environmental exposures, emerging data indicate that the epigenome during the dramatic transformation of male germ cells that occurs in spermatogenesis is also susceptible to environmentally induced epigenetic programming. Nutritional manipulation, such as low-protein diet [74] and prediabetic conditions [75], in adult rodents induces metabolic disorders in offspring through changes in sperm epigenetics of founder male mice. For example, a low-protein diet in adult mice resulted in the downregulation of transcriptional factors and chromatin regulators as well as a decrease in H3K27me3 of specific loci in sperm; however, genome-wide DNA methylation was largely unresponsive to the diet [74]. This latter finding is in contrast to other studies, such that streptozotocin-induced prediabetes conferred widespread alterations to sperm DNA methylation patterns [75]. The susceptibility of chromatin to nutritional manipulation during spermatogenesis is most recently highlighted in work in *Drosophila*, where high-sugar diet in adult males altered methylation of H3K9/K27me3 within chromatin-bound regions of mature sperm that conferred metabolic programming of offspring [76]. Similarly, high-fat diets in adult mice resulted in altered miRNA content [77••] and increased the acetylation of H3K9 in late round spermatids to early elongating spermatids, possibly mediated by a corresponding decreased expression of SIRT6, a stress-response deacetylase [78]. The effect of high-fat diet on global DNA methylation of sperm, however, is inconsistent [77••, 79]

#### Toxicant Exposure

Exposure of adult mice to particulate air pollution obtained from Hamilton, Ontario, increased global methylation of spermatogonia, which persisted through spermatogenesis and remained elevated in mature sperm [80]. Interestingly, these effects were observed after 10 weeks, but not after 3 weeks, of exposure, and persisted for 6 weeks after exposure removal, indicating that the epigenetic modifications occurred in early stages of spermatogenesis (e.g., premeiotic germ cells) [80]. Using a gene candidate approach, chromium(III) chloride exposure to adult mice for 2 weeks decreased sperm DNA methylation of the 45S ribosomal RNA gene [81, 82].

In regard to iDMRs, adult exposure to methoxychlor, an endocrine-disrupting compound, decreased sperm DNA methylation of the paternal iDMR of Meg3 and increased methylation of the maternal iDMRs of Mest, Snrpn, and Peg3 [30]. Similarly, acrylamide exposure for 2 weeks in adult rats decreased sperm DNA methylation of IGF2 iDMR after 35 days, but not after 19 days, indicating that imprinted regions of spermatogonia and primary spermatocytes are susceptible to environmental exposures [83]. These studies demonstrate that the loss and gain of methylation in iDMRs during spermatocytogenesis as previously described [13, 49] can be modified by environmental exposures.

In humans, eight cross-sectional studies in adults to date have documented that chemical exposures, mostly cigarette smoking, are linked with alterations to the sperm epigenome (Table 1). Sperm from adult male smokers exhibited altered miRNA expression [84], higher LINE-1 methylation [85], elevated histone-to-protamine ratios [86, 87], and increased global acetylation of H4K8 and H4K12 [88], compared to sperm from non-smokers, suggesting that chronic smoking exposure may lead to a host of epigenetic changes in the sperm, although imprinted genes H19 and IGF2 were unchanged [89]. In regard to endocrine-disrupting compounds, exposure to perfluoroalkyl substances among a general population study in Europe did not find consistent associations between exposures and global as well as repetitive sequence DNA methylation [90]. Miao et al. (2013) found that urinary BPA exposures were inversely associated with LINE-1 methylation in occupationally exposed workers as well as in non-exposed workers with low exposures. Interestingly, no significant associations were found between urinary BPA and LINE-1 methylation of leukocyte DNA [91•]. This observed decrease in LINE-1 methylation in sperm may have strong public health implications as the occupationally non-exposed workers in the study had lower BPA levels than what has been reported for the US general population [92].

**Table 1:** Summary of the epidemiologic studies of environmental influences on sperm epigenetics in adulthood

Life period	Design	Exposure	Main results	Reference
Adulthood	Cross-sectional	Smoking	25 unique miRNAs showed different expression levels between smokers and non-smokers	[84]
Adulthood	Cross-sectional	Smoking	Before swim up, acetylations of H4K8 and H4K12 sperm cells were statistically significantly increased in smokers compared to non-smokers while no significant changes were observed in the global 5-mC% or acetylation of H3K9, H3K14, H4K5, and H4K16. The sperm cells isolated after swim up revealed no differences in acetylation of any histone or global 5-mC%	[88]
Adulthood	Cross-sectional	Smoking	Heavy smokers showed significantly higher percentage of sperm cells with elevated histone-to-protamine ratios compared to never smokers	[86]
Adulthood	Cross-sectional	BPA	BPA exposure is significantly correlated with lower-sperm LINE-1 methylation among Chinese factory workers, including those exposed to BPA levels equal or lower than reported in the US general population	[91•]
Adulthood	Cross-sectional	Smoking	Smokers showed more abnormal histone to protamine transition compared to non-smokers	[87]
Adulthood	Cross-sectional	Smoking	Smoking is associated with elevated methylation of LINE-1, but not Alu and Sata.	[85]
Adulthood	Cross-sectional	Smoking	H19 and IGF2 methylations were not different between smokers and non-smokers	[89]
Adulthood	Cross-sectional	Perfluoroalkyl substances	No consistent associations between exposure to perfluroalkyl substances (perfluorooctane sulfonate, perfluorooctanoic acid, perfluorohexane sulfonic acid, perfluorononanoic acid) and global or repetitive sequence (LINE-1, Alu, Satα) DNA methylation	[90]

### Sperm Epigenetics and Offspring Development

After fertilization, parental-specific epigenetic marks of gametes undergo reprogramming to establish totipotency in the developing embryo. The kinetics of demethylation differs between parental genomes, whereas the paternal genome is actively and the female genome is passively demethylated [93] (Fig. 1). While it has been widely recognized that parental-specific iDMRs and certain classes of repetitive sequences, such as IAPs, escape this reprogramming event [94], sperm DNA methylation in other genomic loci may also be resistant to reprogramming and also contribute to this non-Mendelian form of inheritance, as demonstrated in the numerous studies discussed in the previous sections. Technical advances in next-generation bisulfite sequencing of small quantities of cells have recently allowed for the resolution of genome-wide methylation maps of mouse gametes and through post-implantation embryogenesis to better understand gamete-specific heritable DMRs. In addition to known iDMRs, one study identified over 1600 CpG island germline DMRs between oocytes and sperm and over half of these were found to be at least partially resistant to demethylation of which 34 were sperm-methylated germline DMRs [95]. Moreover, Meissner and colleagues identified over 4894 sperm-derived DMRs that were enriched in intergenic regions and retained intermediate methylation values during demethylation [96]. Similarly, 34 sperm-derived DMRs identified within CpG islands were also partially resistant to demethylation [95]. However, in both of these studies, these DMRs appear to be targets for de novo methylation after implantation [95, 96]. The relevance of these sperm-derived DMRs in regard to environmental exposures and epigenetic inheritance remains unclear.

Furthermore, the epigenetic inheritance via sperm is not confined to DNA methylation, as other epigenetic factors such as histone retention and non-coding RNA (ncRNA) are likely to act, in concert, to elicit paternal epigenetic inheritance. Since sperm protamines are quickly replaced, within 1 h, by oocyte-derived histones in the zygote [97, 98], the location and modifications of retained histones in the sperm genome likely provide a structural framework to govern reprogramming events within the paternal genome. Similarly, sperm-derived RNAs, including ncRNAs, are proposed to influence embryo development and transgenerational inheritance by providing a window into the environmental history of sperm [99]. For example, paternal stress in adult mice altered sperm miRNA content as well as offspring stress responsivity [100]. Recent data also indicate that sperm-derived factors may not be the only paternal component for proper embryo development, such that ablation of the seminal plasma by surgical excision in mice impaired conception and, among surviving offspring, altered growth trajectory and metabolic parameters [101]. Recently, human seminal exosomes were found to harbor unique profiles of small ncRNAs, including miRNAs, Y RNAs, and tRNAs [102•]. These results indicate that the seminal plasma is not only a transport medium for sperm but contains important non-genetic constituents, such as hormones and exosomes, that act to regulate the female tract environment to support embryo development [103].

## Conclusions

There are numerous epigenetic reprogramming events throughout the life course of the male germ cell, and each may represent a unique window of susceptibility to environmental exposures. Data demonstrate that such inputs from the environment are embodied within the epigenome of sperm and, in turn, are acquired during embryo development. Future animal research needs to expand on these findings by characterizing the full spectrum of sperm epigenetic changes induced by environmental exposures at each window of germ cell development. Additionally, prospective cohort studies are necessary to determine the response of sperm epigenetics in relation to early life environmental exposures. Understanding sperm epigenetics is critical to advance our understanding of paternal environmental determinants of offspring health and development. Such research may result in a paradigm shift in the way reproductive success is viewed, such that the burden of environmental health may not be restricted to expectant mothers but rather is shared with male partners. In this manner, males may need to monitor their environmental health months prior to conception in order to optimize their sperm epigenome for fertilization.
